# No Rest for the Weary: Migrating Songbirds Keep Their Wits without Sleep

**DOI:** 10.1371/journal.pbio.0020221

**Published:** 2004-07-13

Every spring and fall, billions of songbirds fly thousands of miles between their summer breeding grounds in North America and their wintering grounds in the more hospitable climes of southern California, Mexico, and Central and South America. While some birds fly during the day, most, including the white-crowned sparrow, fly under cover of night. Many aspects of this remarkable voyage remain obscure, especially if, and how, nocturnal migrators get any sleep at night.[Fig pbio-0020221-g001]

**Figure pbio-0020221-g001:**
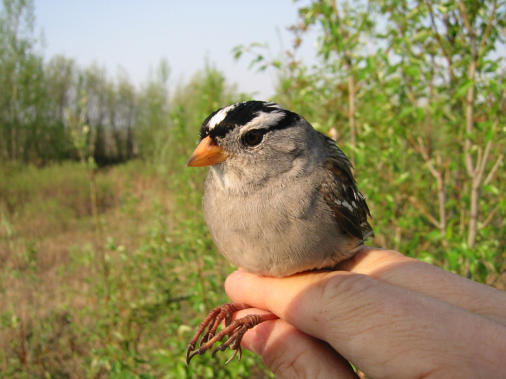
White-crowned sparrow (Zonotrichia leucophrys gambelii)

A tracking study of the Swainson's thrush found that the roughly seven-inch birds flew up to seven hours straight on six of seven nights, racking up over 930 miles. While the study didn't track their daytime behavior, the birds' migratory pace—as well as the increased activity required to sustain migrations—suggests little time for sleep. Yet field observations indicate that presumably sleep-deprived fliers appear no worse for wear, foraging, navigating, and avoiding predators with aplomb. Researchers are left trying to reconcile this observation with the vast body of evidence linking sleep deprivation to impaired neurobehavioral and physiological function. How do songbirds cope with so little sleep? Do they take power naps? Have they taken “sleep walking” to new heights? Or have they managed to selectively short-circuit the adverse effects of sleep deprivation during migratory stints?

To investigate these questions, Ruth Benca and colleagues studied cognitive and sleep behaviors in captive white-crowned sparrows over the course of a year. The sparrows fly nearly 2,700 miles twice a year between their Alaska and southern California homes. In laboratory cages, the birds' migratory instincts manifest as increased restlessness at night during the migratory season, with lots of hopping around and wing flapping.

Niels Rattenborg et al. characterized the birds' activity levels with motion-detection measurements and video recordings, and placed sensors on their brains to monitor their seasonal sleep patterns. The brain recordings showed a marked seasonal difference in both the amount and type of sleep during a 24-hour period: migrating birds spent roughly two-thirds less time sleeping than nonmigratory birds and fell into REM sleep (the dream stage of sleep, marked by rapid eye movements) much sooner. Birds displaying active migratory behavior appeared completely awake during such activity. Cognitive tests—birds performed a task that involved pecking a key in exchange for seed—revealed that birds in the nonmigrating state suffered cognitive deficits when sleep-deprived but displayed an “unprecedented” ability to maintain cognitive function in the face of ongoing sleep loss in the migratory state.

These results suggest that wild songbirds drastically reduce sleep time during migration, though Benca and colleagues concede it's impossible to know for sure without recording the birds in action. And it is unclear what molecular mechanisms jumpstart the migratory mindset. Such an ability to temporarily circumvent the need for sleep, however, could prove useful for humans in situations that demand continuous performance. Some studies link migration with increased neuroendocrine activity, which is in turn associated with sleep disruption, accelerated timing of REM cycles, and mood disorders in humans. “Like migrating sparrows,” the authors note, “both depressed and manic patients show reduced latency to REM sleep, loss of slow-wave sleep, and reduced amounts of total sleep.” Given the parallels between migratory behaviors and bipolar illness, it's possible that similar mechanisms may be involved in both.

Whatever the mechanism, the unprecedented imperviousness of migrating songbirds to sleep deprivation, the authors conclude, clearly warrants further testing. But it also raises interesting questions about the role of sleep, which recent studies suggest is required to incorporate novel perceptions into the brain's memory banks. If this is true, how do songbirds consolidate memories of migratory events with so little sleep? Understanding the mechanisms that power the sleepless flight of songbirds promises to unravel one of the longstanding mysteries of their improbable journey. It may also shed light on the origins of sleep-related seasonal disorders and the much-debated role of sleep itself.

